# Editorial: Ecological distribution, functional diversity, and the biogeochemical cycle of microorganisms in karst

**DOI:** 10.3389/fmicb.2023.1265640

**Published:** 2023-08-11

**Authors:** Xiangyu Guan, Qiang Li, Hongchen Jiang, Werner E. G. Müller

**Affiliations:** ^1^School of Ocean Sciences, China University of Geosciences (Beijing), Beijing, China; ^2^Key Laboratory of Karst Ecosystem and Treatment of Rocky Desertification, Ministry of Natural Resources, Key Laboratory of Karst Dynamics, Ministry of Natural Resources & Guangxi, Institute of Karst Geology, Chinese Academy of Geological Sciences, Guilin, China; ^3^ERC Advanced Investigator Grant Research Group at the Institute for Physiological Chemistry, University Medical Center of the Johannes Gutenberg University, Mainz, Germany

**Keywords:** microbiology community, bacteria, archaea, fungi, virus, diversity of functions, biomineralization, desertification

Karst ecosystems are an important part of the Earth surface system, and contribute significantly to global and regional climate and environmental changes. Karst ecosystems are inhabited by abundant and diverse microorganisms, which are extensively involved in the biogeochemical cycle of elements. Therefore, it is vital to investigate the identity, functions, interactions with environments, and ecological roles of microorganisms in Karst ecosystems. Recently, with the use of new technologies (next-generation sequencing, different omics) and the intersection with other disciplines (such as mineralogy and geochemistry), the research on microbial ecology and biogeochemistry in these ecosystems has made ever-changing achievements in many aspects, such as the formation mechanism of microbial community, microbial interactions, biogeochemical cycle processes of elements mediated by microorganisms and their influence on mineralization and weathering of carbonate rock surface, changes of karst landform caused by microbial action, and microbial involvement in carbon cycle and its environmental impact and feedback. In short, microorganisms play an important role in the material and energy cycles in karst systems. Therefore, the study of microbial community composition and ecological function in karst system has attracted extensive attention. Without knowing the CO_2_ balance in the atmosphere, especially over the karst-rich areas, like in Spain or in China, it is not possible to set up a reliable prediction of eco-commercial predictions.

In this Research Topic, 13 articles are here included, 10 and 3 articles on soil and water environments in karst ecosystems, respectively. We are grateful to all authors who contributed to this Research Topic. We are also grateful to all reviewers, handling editors and editorial staff who contributed during the editing and article production processes.

Among the articles related to studies on karst soil environments, Cheng et al. characterized and analyzed microbial communities, keystone taxa and the predicted ecological functions from three niches including weathered rock, sediment, and drip water inside the Heshang Cave (Central China) and three types of soils overlying the cave (forest soil, farmland soil, and pristine karst soil). Li Q. et al. investigated microbial structure patterns from bulk soil in A layer (0–10 cm) and B layer (10–20 cm) along altitudinal gradients in one karst graben basin of Yunnan-Kweichow Plateau. Jiang et al. reported that the structure, keystone taxa, assembly mechanism of bacterial and fungal communities and their relationships with environmental factors in one unique karst Tiankeng environment (the world's deepest sinkhole and the largest in the Shaanxi cluster, located in Fengjie County of Chongqing, southwestern China). Chen et al. reveal that soil nutrient content differences play an important role in regulating the cyanobacterial diversity and composition for further research and application of soil ecological restoration of cyanobacteria in biological soil crusts of karst desertification areas (in the Guizhou Plateau, southwestern China). Zhang et al. found that soil types played a predominant role in shaping rhizosphere microbial communities in northern tropical karst and non-karst seasonal rainforests. Sun et al. proposed that the different variations in microbial communities during bamboo invasion may be related to the influence of invasive bamboo on the soil properties such as pH, contents of organic matter and total phosphorus at different invasion stages. Hu et al. indicated that the magnesium-modified citrus peel biochar inhibited the organic carbon mineralization in citrus orchard soils and was more favorable to the increase of soil organic carbon fraction. Pu et al. investigated the effects of mixed application of different ratios of N fertilizer and green manure on the soil microbial community and rice yield in one karst paddy area, and found that the combined application of N fertilizer and green manure reduced the complexity of soil microbial network. On the basis of the rice yield, the authors recommend that nitrogen application should be reduced by 20–40% for rice production in ecologically sensitive karst areas. Li Y. et al. investigated soil chemical properties and microbial community stability in karst mountain soils, and showed that organ mineral fertilizer could replace chemical fertilizers or common organic fertilizers in terms of improving soil fertility and increasing crop yield and quality. Ning et al. showed that bacteria including coliforms can survive for a long time in karst soils overlying karst rocks and were unable to prevent bacteria from infiltrating into groundwater.

Among the articles related to studies on karst water environments, Liang et al. disclosed that microbial activity or the input of anthropogenic acids potentially affect carbonate dissolution, eventually altering the hydraulic properties of karst aquifers. Guan et al. revealed that there were significant annual and seasonal changes in the physicochemical properties and microbial communities of karst river, and antibiotics and inorganic nitrogen pollution indirectly affected the cycles of nitrogen and sulfur elements through microbial ecological modules. Zhong et al. found that in karst groundwater, the community assembly of a few abundant taxa was shaped by deterministic processes, especially homogeneous selection, while that of a large number of rare taxa was controlled by stochastic processes.

The collection of papers on the important karst terrestrial and biotic cycles will surely contribute to a further awareness of those beautiful, natural and biotic communities.

We are delighted to publish this Research Topic in Frontiers in Microbiology. We hope that this Research Topic will be interesting and useful to the readers of the journal, and broaden the knowledge of karst environment. The findings presented in this Research Topic are exciting, but still limited. In the future, the application of innovative research technologies and intensive and in-depth international collaboration will undoubtedly unveil more exciting aspects of karst ecosystem. Based on the data given in the papers research on karst biocoenosis will achieve and surpass new frontiers.

## Author contributions

XG: Writing—original draft. QL: Writing—review and editing. HJ: Writing—review and editing. WM: Writing—review and editing.

